# Examining the effect of perceived performance-contingent gains, losses and errors on arithmetic

**DOI:** 10.1371/journal.pone.0249696

**Published:** 2021-04-08

**Authors:** Ram Naaman, Liat Goldfarb

**Affiliations:** E.J.S. Brain Research Center for the Study of Learning Disabilities, University of Haifa, Mount Carmel, Haifa, Israel; Educational Testing Service, UNITED STATES

## Abstract

Gains and losses have previously been found to differentially modulate Executive Functions and cognitive performance depending on performance contingency. Following recent findings suggesting that random gains and losses modulate arithmetic performance, the current study aimed to investigate the effect of perceived performance-contingent gains and losses on arithmetic performance. In the current study, an arithmetic equation judgment task was administered, with perceived performance-contingent gain, loss, and error feedback presented upon each trial. The results from two experiments suggest that when perceiving gain and loss as performance-contingent, the modulation of arithmetic performance, seen previously under random contingency conditions was entirely eliminated. In addition, another type of feedback was examined in the context of an arithmetic task: post-error adjustments. When performance after error feedback was compared to performance after other aversive performance feedback such as loss signals, only errors, but not other aversive feedback, modulated performance in the subsequent trial. These findings further extend the knowledge regarding the influence of gain and loss situations, as well as errors, on arithmetic performance.

## Introduction

Serving as a cornerstone for various higher branches of mathematics, arithmetic is often regarded as a fundamental body of knowledge acquired during our school years [1]. It includes the basic mathematical operations of addition and subtraction or multiplication and division. Apart from serving as one of the foundations of higher mathematics, arithmetic takes a crucial role in our life in a variety of daily situations. Performing arithmetic was found to involve a variety of cognitive mechanisms, both general and domain specific. Among those, executive functions (EFs) were found to play a crucial part in many proper arithmetic performances [2–4].

The term EFs refers to a wide array of cognitive control mechanisms responsible for regulating goal-directed behavior, especially in novel or demanding situations [5, 6]. These mechanisms, such as prioritizing, working memory (WM), shifting (i.e., switching or cognitive flexibility), and inhibition, alongside other mechanisms such as immediate recall of numerical information, were found to be heavily involved in arithmetic performance [3, 7–10].

Some of the most frequently studied EFs in the context of arithmetic performance are WM, inhibition, and shifting. The reliance on these EF mechanisms seems to grow as a function of the calculation’s level of complexity. For example, increased complexity as a result of multiple intermediate processes involved and their numerical magnitude [8, 11–14], calculation procedures requiring carrying and borrowing, switching between arithmetic operands or between multiple calculational steps, or performing multiple addend calculations [12, 14–16].

While arithmetic calculations are routinely performed in our everyday life they are also very often accompanied by positive or negative performance feedback. Imagine dining in a restaurant with friends when the bill is presented, which now must be split between those present. While fast and accurate calculation might reward you with compliments from your surroundings, slow or inaccurate calculation will probably result in negative feedback such as sarcasm or even joking at your expense. Similarly, positive and negative feedbacks are also very present in the educational systems as students’ performance is continuously evaluated either formally (e.g., exams or paper submissions) or informally (e.g., when a child is asked a question during class they can be rewarded for a correct answer or publicly corrected in front of their classmates).

Whether in class as students or in various daily situations, receiving feedback for our arithmetic performance is very common. Nevertheless, the research in this specific field is relatively scarce. In fact, studies examining the relationship between arithmetic performance and feedback can be classified as: a) studies examining the effect of performance feedback on mathematical performance, b) studies examining the effect of calculation errors on math performance.

### The effects of gain and loss on arithmetic performance

While very little research has been devoted to examining the effect of feedback specifically on arithmetic performance, it has been widely established that various affective signals are involved in regulating cognitive performance. Specifically, the influence of gain and loss (or rewards and punishments) on executive functions was frequently studied under different cognitive contexts and tasks. These include the effect of gains and losses on the ability to effectively maintain task focus [17–19], adjust to changing task requirements [20], inhibition and attentional processes [21].

Although studied intensively, the modulation of gain versus loss on these various cognitive performances seems to differentiate across studies and experimental designs [for a review, see 22, 23]. One important factor in this differentiation seems to be the administration contingency. That is, when the stimulus is perceived as either performance-contingent (i.e., perceived as related to participant’s performance) or randomly administered (i.e., perceived as not related to participant’s performance). The literature suggests that perceiving gain and loss stimuli as performance-contingent (i.e., depending on performance) might differentially affect cognitive performance when compared to stimuli perceived as randomly administered. For example, when participants were told that stimuli were administered in a random manner, gain stimuli were found to hinder various cognitive mechanisms such as inhibition, adaptation to conflict, and WM in some studies [21, 24, 25], and to facilitate cognitive stability and WM in others [15, 25, 26]. On the other hand, studies that investigated how gain and loss stimuli affect behavior when they are perceived as performance-contingent, found a facilitative effect of gain stimuli on WM processes, proactive control, and conflict adaptation in some cases [20, 27–29], and a hindering effect on cognitive stability and complex problem solving in others [26, 30].

The differences between performance-contingent versus random reward administration contingencies are reflected by the perceived causal link (or the absence of such link) between performance and expected outcomes (i.e., gains and losses). As such, they can also be viewed in terms of efficacy. In other words, efficacy relates to "… the core believe that one has the power to produce effects by one’s actions" [31]. In a recent study, the effect of efficacy and rewards on cognitive performance was tested using a stroop task paradigm [32]. In this study, reward sizes were manipulated (large versus small gains) along with the level of efficacy (random versus performance-contingent). Prior to each trial, participants received a cue representing the next trial’s contingency and potential reward size and following their response they received a feedback representing the amount of bonus won. Across three experiments, significant effects were found for reward sizes and efficacy with participants performing faster after large reward cues and performance-contingent ones when compared to small rewards and random administration. Interestingly, reward size and efficacy interacted suggesting that participants performed fastest in under the larger rewards that were given with accordance to their performance when compared to large rewards that were given randomly. The authors concluded that efficacy modulate the tradeoff between the perceived cost of allocating more control resources to the task at hand and the potential rewards expected from better performance [33]. This modulation of administration contingency on cognitive control performances, as represented by the efficacy levels, was also found by several others with similar findings [34, 35].

One possible methodology for examining how perception of feedback as performance-contingent influences behavior is by administering the feedback stimuli in accordance to the participant’s actual performance. Nevertheless, this method results in imbalanced stimuli administration, since feedbacks will be presented unequally across trials and experimental conditions. Using this methodology obligates several adjustments to overcome these obstacles (e.g., staircase algorithms), thus making it harder to compare to random contingencies where stimuli administration is controlled and balanced across trials and conditions [26, 28, 29]. Another possible methodology for examining performance-contingent feedback is by administering the feedback randomly and equally across trials and conditions while creating the impression of performance-continent feedback among participants. This way, while administration is random, carefully controlled, and balanced, participants perceive it as performance-contingent [36].

Some studies directly examined the effects of performance-contingent versus random gain and loss stimuli. In a study by Braem et al., these effects were examined in a conflict task-switching paradigm [26]. According to their results in the performance-contingent condition gain stimuli enhanced cognitive flexibility while loss stimuli led to enhanced cognitive stability (as measured by the switch cost following congruent versus incongruent trials). Contrarily, their results in the random condition suggested the opposite, with gain stimuli leading to stronger cognitive stability while loss stimuli led to stronger flexibility (see also [37]).

Contrary to the results of Braem et al., a different pattern of results was obtained in a study by Chiew and Braver [28]. In their study, random emotional stimuli and performance-contingent rewards were used in order to compare the effects of performance-contingent rewards versus random emotional stimuli on cognitive control using an AX-continues performance task (AX-CPT). The AX-CPT tasks is considered to represent sustained attention and context processing as well as pro-active and re-active control processes. In this task, participants are required to respond to a sequence of cue and probe letters. Specifically, a predetermined target response is required following the probe letter "X" but only when preceded by the cue letter "A" (AX trials). These target trials are usually presented with high frequency while non-target trials ("X" following another letter then "A", "A" followed by another letter then "X" or other combinations) are presented with substantially lower frequency [17]. Their results suggest that while both contingencies tend to promote cognitive stability, performance-contingent rewards led to a substantially stronger effect.

In partial support in this direction a recent study by Park et al., compared emotional (non-monetary) and motivational (monetary) rewards on both behavioral measures and neural activity using neuroimaging [38]. Interestingly, their behavioral findings suggest similar facilitation effect for either symbolic or value dependent positive stimuli (i.e., positive emotion or reward) when compared to negative ones. Nevertheless, their neuroimaging findings are not conclusive as for the existence of shared neuronal mechanisms underlying both stimuli types.

In an attempt to disentangle the affective cognitive interaction, Notebaert and Braem suggested a new conceptual framework [39]. According to their proposal, reward includes three distinguishable constructs: affective (hedonic), learning, and motivational. The administration of random affective gain stimuli (e.g., smiley faces, positive pictures, and substantial monetary gains given irrelevantly of participants’ performance) lead to a general hedonic affect. This affect, in turn, elicits a cognitive "exploration" mode resulting in enhanced cognitive flexibility. Carver [40] suggested that this shift towards cognitive flexibility following positive affect could be the result of signaling that "things are going better than necessary" and therefore allowing the cognitive system to lower the resources invested in the task at hand and thus allowing more flexible responses to unforeseen events (but see, [26]).

Contrary to random gains, performance-contingent rewards (e.g., verbal feedback and neutral signals representing small monetary rewards) tap the "learning" construct of reward. This term is borrowed from the field of reinforcement learning and refers to the increased likelihood of observing the behavior preceded by the reward. According to Braem and Notebaert’s model, activation of the learning construct results in an "exploitation" mode, enhancing cognitive stability. Specifically, they suggested that performance-contingent rewards strengthen task relevant associations allowing better task-focus and therefore better performance.

It is important to note that both cognitive stability and flexibility are essential mechanisms through which efficient regulation of behavior takes place. Therefore, while shifting towards flexibility might facilitate performance when attending to changing task characteristics (e.g., task switch paradigms), it could hinder performance under the opposite task characteristics (e.g., AX-CPT, flanker, and Stroop [41]).

In the context of arithmetic performance, only two studies examined the effect of affective stimuli on performance and both used a random rather than performance-contingent stimuli. In a study by Naaman and Goldfarb [15], the influence of random gain and loss stimuli on arithmetic performance was examined. In three experiments, participants solved arithmetic equations significantly faster after gain stimuli, suggesting a facilitative effect of gain stimuli on arithmetic performance when compared to loss. In a recent study by Kulkova and Fischer, a similar pattern of results was obtained, according to which positive stimuli facilitated arithmetic performance [42].

Since, as noted above, the perception of gain and loss stimuli as random or performance-contingent can differentially modulate performance, a new question arises concerning the modulation of performance-contingent gain and loss stimuli on arithmetic performance and its direction. Considering the previous results from the random contingencies studies and the theoretical framework described above, if random gains previously found to facilitated arithmetic performance through the promotion of more flexible cognitive environment, performance contingent gains might lead to the opposite and hinder performance (e.g., slower RTs). Nevertheless, unlike other cognitive tasks such as task switch paradigms, stroop or flanker, arithmetic encompasses multiple cognitive processes and therefore performance can be modulated in multiple ways along the way. For example, while more complex arithmetic equations require EFs (e.g., WM, inhibition, flexibility) to a greater extent, simpler equations might benefit from greater task focus (i.e., enhanced stability). Moreover, the shifting between these different types of arithmetic procedures itself (simple versus complex or two addend equations versus three addend ones) might also require flexibility for more efficient performance. Considering all of the above, while we hypothesized that perceived performance-contingent gains and losses will differentially modulate performance when compare to random stimuli, the direction of this change remain relatively unclear. Therefore, the first aim of the current study will be to examine the effect of perceiving gain and loss stimuli as performance-contingent on arithmetic performance.

### The effects of error feedback on arithmetic performance

Aside from gain and loss stimuli, error feedback was also found to serve as an important factor in modulating cognitive performance. Errors are considered a powerful signal, eliciting physiological changes (e.g., changes in skin conductance, heart rate, and startle effect, [43–46]) alongside cognitive and behavioral modulations (e.g., changes in brain activations, [47–49]), performance hit rate, and reaction time [50, 51]. Moreover, errors are assumed to represent emotionally and motivationally salient events. Specifically, they are perceived as threatening, aversive signals that result in the activation of defensive motivational systems and modulation of behavior accordingly [29, 43, 46, 52–54]. In other words, we perceive errors as aversive and threatening events and, as a result, we invest significant effort, referred to as post-error adjustments, in order to modulate our behavior accordingly. One of the most prominent behavioral findings in the context of post-error adjustments, is the significant increase in reaction time (RT) following the commission of erroneous responses, referred to as post-error slowing (PES) [55]. While PES itself is considered a robust effect, various explanations have been offered to account for it. According to the conflict monitoring account, errors or conflicts lead to an increased response threshold [56]. That is, after an error, adaptive control mechanisms are recruited in order to improve performance and prevent future errors by slowing response time. These control mechanisms are thought to change the accuracy vs. RT tradeoff towards a more conservative balance (i.e., we become more cautious), resulting in enhanced accuracy after errors [44, 56–59]. Nevertheless, findings from various studies do not fit this explanation. Specifically, not only do some studies not find significant changes in accuracy following an error [60, 61], others even present evidence of the opposite [49, 50, 62, 63]. In an attempt to better account for the robust findings of PES on the one hand, and the inconsistent findings regarding the accuracy rate on the other, another theoretical explanation was suggested. According to the orienting account, errors are perceived as infrequent events that divert attention from the task at hand, slowing down the processing of task relevant information and resulting in hindered performance in the subsequent trial [51]. While the conflict monitoring and orientation of attention are the most prominent theoretical accounts for PES, other accounts have been suggested in recent years with no clear cut decision [63, 64].

As in the case of feedback and arithmetic, only few studies have examined PES in the context of arithmetic performance. A study by Desmet et al. used an arithmetic multiplication verification task to examine the effect of PES in arithmetic [58]. Their findings show a significant PES effect as well as increased accuracy rates after errors. That is, responses after errors were slower than after correct responses and accuracy rates after errors increased, suggesting that participants might have adjusted their performance strategies upon committing an error.

In another similar study by Van der Borght et al., participants’ solution strategy was also recorded in each trial [65]. According to their results, PES accompanied by decreased accuracy was observed only in post-error trials with no change in solution strategy, whereas no PES and accuracy increase were observed in post-error trials where a strategy change was made.

In a third study, a serial mental addition comparison task [66] was examined. According to the results, while PES was found across all groups, it was most prominent in the high accuracy group compared to the other two groups. Interestingly, contrary to the study of Desmet et al. [58], in this study accuracy rate decreased after errors, with the sharpest decrease in the higher accuracy group of participants when compared to the other two groups. Finally, two recent studies examining post-error adjustments in arithmetic both resulted in a similar pattern of results, with slower RTs and decreased accuracy rate after errors when compared to correct responses [50, 62]. While it seems that most findings in arithmetic tasks converge towards a similar pattern of decreased accuracy and longer RTs after errors, there is no clear cut conclusion as to the most suitable theoretical explanation [49–51, 58, 60, 63, 66].

### The current study

First, the current study aims to address, for the first time, the question of whether and in what direction gain and loss stimuli affect arithmetic performance when perceived as performance contingent. This is important since, as noted above, studies suggest a differential impact of performance-contingent versus random gain and loss stimuli on cognitive performance. Furthermore, the current study will also examine these effects with relation to the influence of post-error slowing (PES) on arithmetic performance. Considering the aversive valence of errors, we also aimed to examine the relationship between the aversive stimuli—the loss stimuli, and PES in the context of an arithmetic task.

In order to examine these questions, we conducted two experiments. In Experiment 1, an arithmetic equation judgment task was administered similarly to that used by Naaman and Goldfarb [15]. Upon each correct answer, a random line drawn face appeared on the screen, representing either monetary gain, loss, or no monetary meaning (i.e., happy face, sad face, or neutral face, respectively). Although the faces appeared randomly and irrelevantly to participant’s RT, participants were informed that their performance in each trial would be measured and compared to a norm, with smiley faces appearing after correct and faster than average responses, sad faces after correct but slower than average responses, and neutral faces after correct responses within the averaged norm. Participants in the current study were instructed on the administration of error feedback following inaccurate responses (i.e., while accurate responses are followed by a line drawn face, inaccurate ones are followed by an error feedback). Experiment 2 was similar to Experiment 1, with a few minor changes in which another neutral condition (an empty circle with no facial features) was added, representing no monetary gain or performance feedback. In sum, the current procedure was identical to the one used in the previous work of Naaman and Goldfarb (further described bellow) with a few minor changes. Unlike the previous study, where participants were aware that the gaining stimuli (smiley faces) appeared randomly and irrelevantly of their performance (i.e., each could appear after a fast response or a slow one, a correct response or erroneous one), in the current study, the gaining stimuli appeared randomly after correct responses only (irrelevantly of RT) but participants were instructed that the presentation of faces was contingent to their actual RT. Additionally, the current study also included "ERROR" signals after erroneous responses. That is, after incorrect responses, no faces administered and instead an error message appeared on the screen.

## Experiment 1

Considering previous findings suggesting gain and loss might differentially modulate performance depending on them being perceived as performance-contingent or random [26, 28, 67], the purpose of Experiment 1 was to examine whether gain and loss stimuli modulate arithmetic performance when perceived as performance-contingent.

## Materials and methods

The study was approved by the ethics committee of the University of Haifa, Israel, and conformed to its standards. Written informed consent was obtained from all participants.

### Participants

Thirty students from the University of Haifa participated in Experiment 1 (mean age = 25.64; SD = 2.75; 20 women). Participants were either paid $7 or received course credits in return for their participation. Participants were also informed they could earn a bonus of up to 2$ depending on their task performance. The inclusion criteria for participation in the experiment was being native Hebrew speaker with no diagnosed learning disabilities or attention deficits. Furthermore, participants performing above 15% error rate in the arithmetic task were excluded from the final analysis (overall 3 participants or 10%). Finally, upon completion of the entire experimental procedure, each participant was questioned regarding his estimation for his performance ("how much do you think you earned?") and then briefed regarding the true random nature of the gain and loss administration (during which they were directly asked if they suspected the experimental procedure was actually random). Participants claiming at any stage they suspected that the gain and loss stimuli were presented randomly and were not dependent on their performance were excluded from the study (overall, two participants or 6%). To sum up, of the initial 30 participants, 25 were included in the final analysis.

### Stimuli

**Fig 1:**
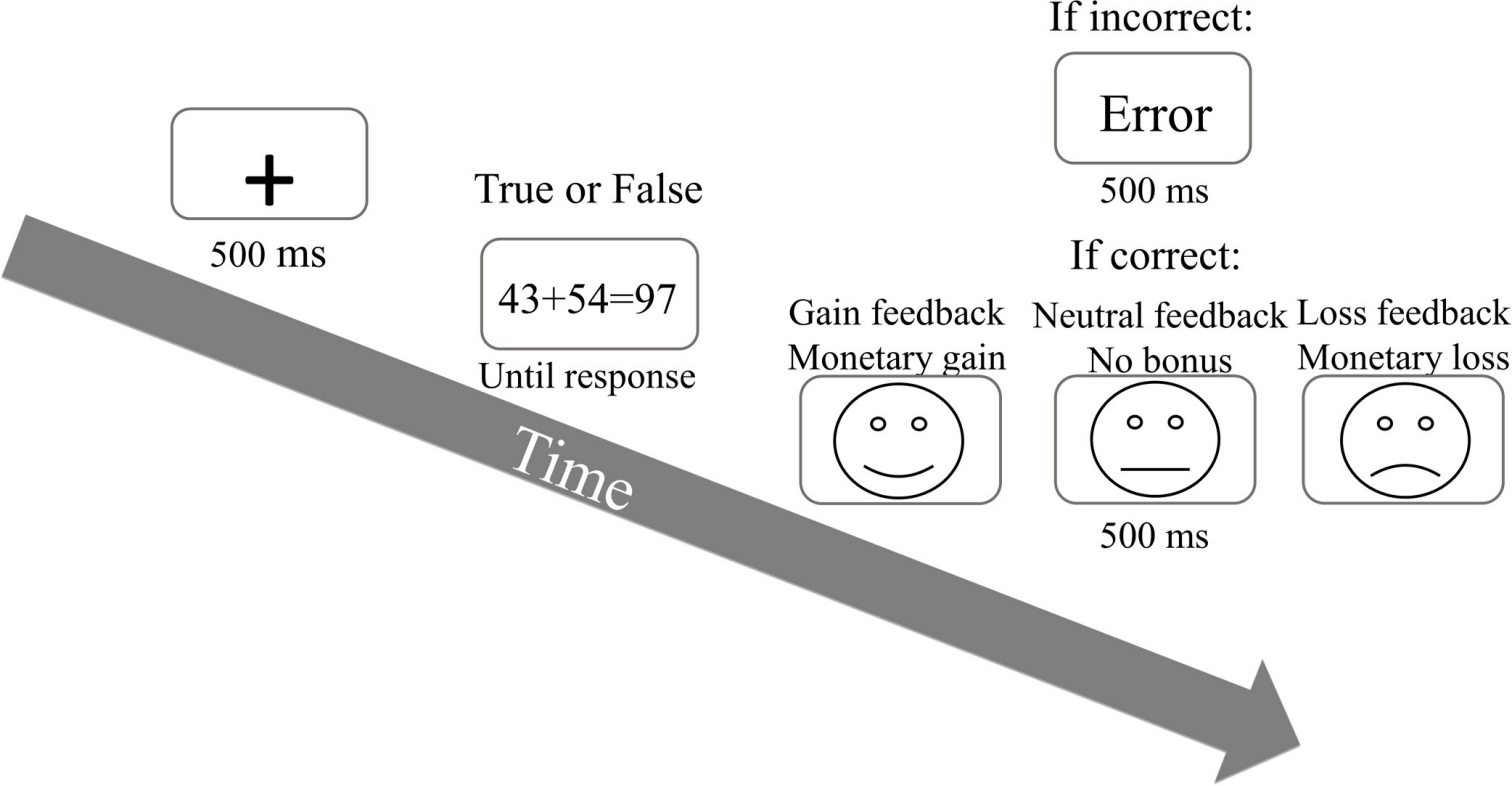
Experiment 1 trial procedure. In each trial, an arithmetic addition equation was presented, and participants were instructed to calculate and judge if the equation’s sum is true or false. Correct trials were followed by one of three line drawn faces, while erroneous responses were followed by an error feedback ("טעות" in Hebrew).

The arithmetic task used in the current study was identical to that used by Naaman and Goldfarb [15], consisting of 266 double-digit arithmetic equations. All equations were presented in the center of the computer screen using Arial font, size 26. Equations were also identical to those in the previous study by Naaman and Goldfarb, with the following guidelines: sums of all equation ranged from 61 to 98; no multiples of 10 or ties were included as addends or sums; no identical addends were used in a single equation. Furthermore, each sum could be the result of either a 2 or 3 addend equation (e.g., 54+35 = 89 or 16+52+21 = 89). Following each equation, a black line drawn face 4.5 cm in diameter appeared in the center of the screen on white background. By manipulating the angle of the curve on the line dawn faces’ mouths, happy, neutral and sad faces were created representing the different gaining conditions. That is, the gaining condition included the different line drawn face feedbacks. This trial procedure is described in Fig 1. It is important to notice that although the trial process described in Fig 1 present the feedbacks following the participants’ performance, the analysis was actually conducted on trial *n+1*, linking each feedback in trial *n* to the RT performance in trial *n+1* (see Fig 1).

### Procedure

The experimental task was programed using E-Prime software [E-Prime 3.0, Psychology Software Tools, Pittsburgh, PA] and administered on an *hp Compaq* computer (Intel i7 core processor) with a Samsung LCD SyncMaster SA350 27 inches monitor (screen resolution: 1920X1080). All participants were tested individually and sat approximately 60 cm from the computer monitor. Participants’ responses were conveyed using the computer keyboard. Every experimental session started with instruction presented on the screen and lasted 20 approximately minutes.

The current procedure is also a modification of that employed by Naaman and Goldfarb [15]. Correct responses were followed by gain, neutral, or loss stimuli, indicated by line drawn faces, but those were presented randomly and irrelevantly of participants’ RT. However, participants were informed that upon each correct answer a line drawn face would appear, depending on how fast they reacted compared to a "norm based on the performance of previous participants". Specifically, participants were instructed that responses that are both correct and faster than average would be followed by a happy face (indicating a gain of $0.15), responses that are correct but within the range of the "norm" would be followed by a neutral face (neutral condition with no monetary meaning), and correct responses slower than the "average" would be followed by a sad face (indicating a loss of $0.15). Finally, participants were informed that incorrect responses would be followed by error feedbacks ("טעות"- “error” in Hebrew) and therefore will have no monetary meaning. While attaching both, "social" valenced faces (e.g., smiley or sad faces) with monetary rewards (earning or losing bonus money) is well documented in the literature [24, 68–72], this combination of stimuli type was found to have an additive effect on cognitive and behavioral performances [73]. Nevertheless, in the current study, this possible additive effect was not examined since the main aim was to examine perceived performance-contingent environment that is as similar as possible to that of the previous random study by Naaman and Goldfarb [15].

Each trial began with a presentation of a fixation cross for 500 ms, followed by an arithmetic equation. The equations were presented with either a correct or incorrect sum. Equations were presented with no time limitation and until a response was conveyed by the participant. Each correct response was immediately followed by a line drawn faces for 500 ms, after which the next trial started, while each incorrect response was followed by an error feedback presented on the screen for 500 ms. Participants were required to respond as fast and as accurate as possible, judging whether if the sum presented for each equation is the correct or incorrect sum. Responses were conveyed using the computer keyboard with the "M" key for correct equations and the "C" key for incorrect ones. Response time and accuracy for each participant were both measured by the computer.

Similar to Naaman and Goldfarb [15], here too the arithmetic task included three blocks. The first block included six equations only and served as a practice block. Upon completion of the first block, the second block started. This block was the experimental block containing a total of 240 equations. 50% (120) of the equations in the second block were easier two addend equations and the rest 50% were more difficult three addend equations. In both the two and the three addend equations, 50% (60) of the equations were presented with the correct sum and the rest were presented with the incorrect sum (50%). Upon the completion of the second block, a third block was administered. This block only included 20 equations and its purpose was to serve as a filler block with overrepresentation of gains resulting with all participants to win the maximum bonus money.

Equation’s difficulty (two addend or three addend equations), equations correctness (correct or incorrect sum) and gaining condition (happy, neutral or sad) were counter-balanced and randomized across the experimental block. Upon completion of the experiment, participants were informed of the experimental procedure and the randomized appearance of the gain and loss stimuli, regardless of their RT.

## Results and discussion

### Effects of gain and loss on arithmetic performance

Only trials from the experimental block were included in the final analysis. Errors, post error trials (total of 15.6%) and trials with outlier RTs (+/- 2 standard scores and under 300 ms; total of 3.63%) were excluded. It is important to note that while post-error trials were excluded during the analysis of the effect of the different gaining conditions on performance RT, they were again included to analyze the effect of post-error slowing (PES). Considering that the feedback stimuli were presented following participant’s response, we therefore analyzed the performance RT in trial *n+1*. That is, we examined how the presentation of each feedback modulated performance in the next trial. The prevalence of each feedback stimulus across participants was almost identical with 33.237% (n = 1733) gain trials, 33.410% (n = 1742) neutral trials, and 33.352% (n = 1739) sad trials. Finally, mean RT under the different conditions was calculated for each participant, and a repeated measures three-way ANOVA was performed, with gaining, equation difficulty, and equation correctness as within subject independent variables and RT (*n+1*) as the dependent variable. The ANOVA was performed using IBM, SPSS statistics software 25.

**Fig 2:**
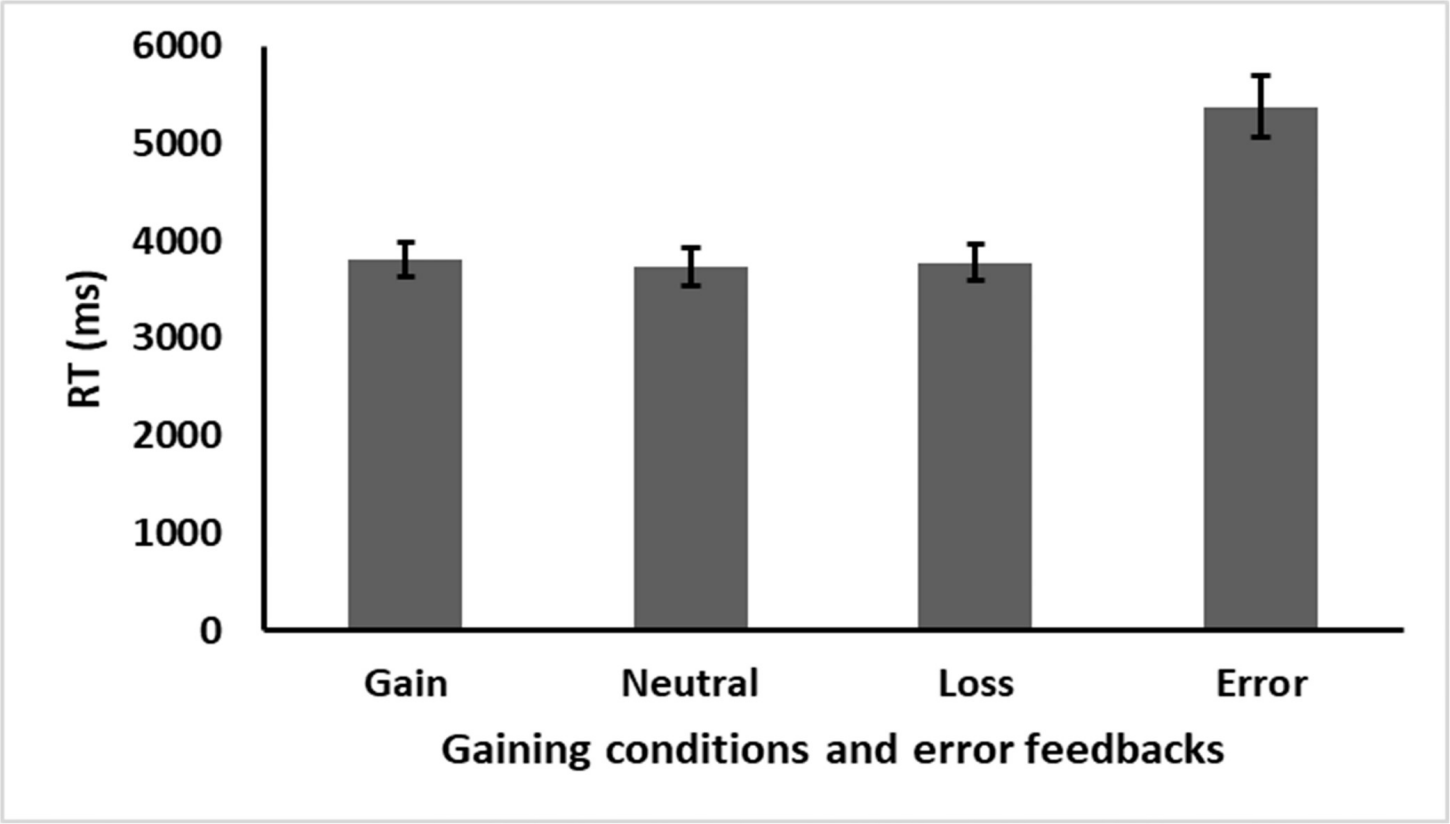
Experiment 1 results. Performance after the different gaining conditions (performance in trial n+1). Error bars represent standard error.

A significant effect was found for equation difficulty, *F*(1,24) = 269.933, *p* < .001, η_p_^2^ = 0.918. participants solved the more difficult three addend equations slower the easier two addend ones. It also interacted with equation correctness, *F*(1,24) = 22.271, *p* < .001, η_p_^2^ = 0.481. Most importantly, gaining condition (i.e., gain, neutral, or loss) failed to reach significance levels, *F*(2,48) = 2.738, *p* = .074, η_p_^2^ = 0.102 (see Fig 2). That is, no differences in performance RTs were observed between the different gaining conditions. No other main effects or interactions were found, all *p*s > .05.

Further analysis of the gaining condition main effect revealed no differences in RT after gain stimuli when compared to loss stimuli, *t*(24) = 0.877, *p* = .388, *d = 0*.*175*. Examining the accuracy rate under the different gaining conditions resulted in no significant effects *F* < 1.

The non-significant findings regarding the effect of performance-contingent gain versus loss stimuli on arithmetic performance could be the result of either a true null hypothesis (i.e., performance-based gain stimuli do not facilitate arithmetic performance when compared to loss) or data insensitivity (e.g., noise). Since orthodox statistical methods do not allow to distinguish between the two, a Bayesian statistical analysis was conducted.

Bayes factors (BF) for the ANOVA comparisons were calculated using JASP statistical software 0.9.2, with the default prior values [74, 75]. Analyzing the gaining condition’s main effect on RT revealed a BF_10_ of 0.038, suggesting moderate evidence for the null hypothesis (i.e., no differences between the different gaining conditions). In order to further examine the effect of gain and loss, another Bayesian analysis was conducted using the Dienes online Bayes calculator [76] and thus enabling us to easily analyze the current data while using different priors from previous data sets [77–79]. This time, the data from the previous study of Naaman and Goldfarb [15] served as prior values to test our hypothesis with prior mean RT after gains of 4008 ms (SD = 1261, df = 41) (see S1–S4 Datas for the entire data set). Comparing the RTs after loss to the RTs after gain stimuli using previous data priors with 95% highest density distribution (HDI) supported our hypothesis, BF_10_ = 0.12 (95% HDI = 0.003–0.329) suggesting there are no differences in RT after gain when compared to after loss stimuli.

### Post-error slowing (PES) in arithmetic performance

In order to analyze the error rate and post error effects on performance, we again analyzed the data including the post-error trials as mentioned above. Here, the prevalence of errors was 7.44% (n = 443) across all trials. As predicted a PES effect was found, with participants solving arithmetic equations significantly slower after committing an error compared to after correct trials, *t*(24) = 7.886, *p* < .001, *d* = 1.577, BF_10_ of 348206 (considered extreme support for the alternative hypothesis). Nevertheless, post-error and post-correct trials did not result in differences in accuracy, *t*(24) = 1.062, *p* = .299, *d* = 0.212, BF_10_ of 0.35.

### Comparing the effect of errors versus aversive stimuli on arithmetic performance

The effect of error feedback was further compared with other negatively salient feedback stimuli. The analysis revealed a significant main effect for the different feedback conditions, F(3,72) = 97.102, p < .001, η_p_^2^ = 0.102, BF_10_ of 5.497e+21 (considered extreme support for the alternative hypothesis), for differences in RT as a result of the different feedback conditions. Further analysis revealed that participants solved arithmetic equations significantly slower after errors when compared to after loss stimuli, *t*(24) = 9.643, *p* < .001, *d* = 1.929, BF_10_ = 1.120e+7 or neutral stimuli *t*(24) = 10.631, *p* < .001, *d* = 2.126, BF_10_ = 6.784e+7 (Both BF values represent extreme support for the alternative hypothesis).

To sum up, we aimed to examine the effect of gains, losses and errors on arithmetic performance in a perceived performance-contingent environment. Interestingly, results show that when perceived as performance-contingent, gain stimuli did not differentially affect arithmetic performance when compared to loss. Furthermore, errors led to slower RTs (PES) both when compared to post correct-trials or when compared to other negatively salient feedbacks (i.e., loss stimuli).

## Experiment 2

The purpose of Experiment 2 was to further replicate the results of Experiment 1 while adding another neutral condition and further examining the relationship between the gain, loss and neutral stimuli to the error feedbacks.

## Materials and methods

**Fig 3:**
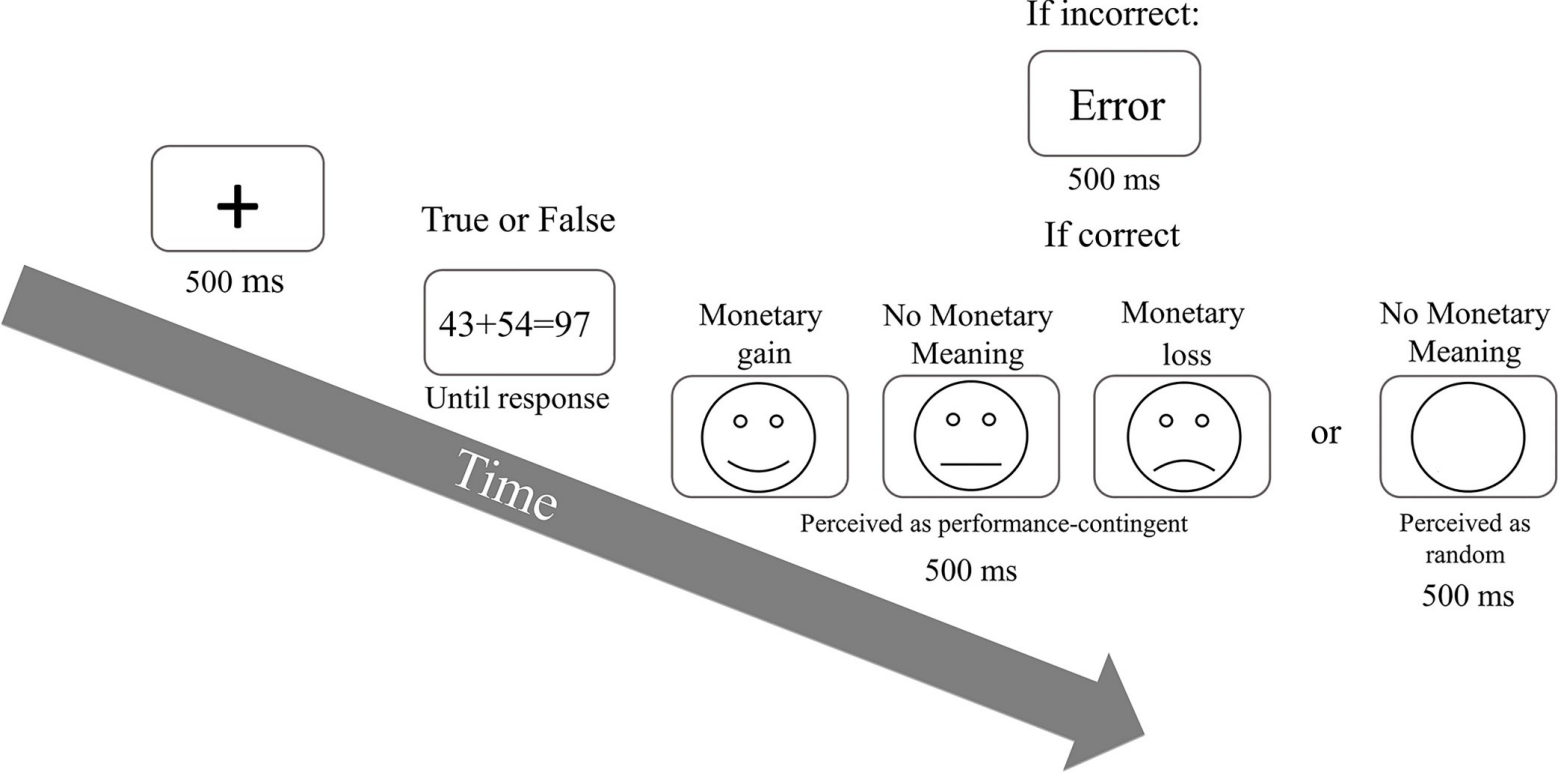
Experiment 2 trial procedure. Experiment 2 included a fourth gaining stimuli, comprised of an empty circle with no facial features. This stimulus appeared randomly after correct trials and had no monetary meaning (i.e., neutral non-feedback). again, here too performance after each gaining condition (*n+1*) is presented.

In Experiment 2, a similar method was used as in Experiment 1 with the exception of the following: Of the 26 students participating (mean age = 25.31; SD = 3.13; 19 women), 22 were included in the final analysis (two or 7.7% participants were excluded because of higher than 15% error rate and another two were excluded because they figured the feedbacks were administered randomly). In Experiment 2 a fourth stimulus was added to the gaining condition. This stimulus was comprised of an empty circle identical to the line drawn circles of the emotional face stimuli, though with no facial features inside (see Fig 3). Both neutral stimuli (i.e., the neutral feedback and the neutral non-feedback) had no monetary meaning and they differed only in their perceived contingency. That is, while the regular neutral face stimuli were perceived as performance-contingent (e.g., performing within the norm), participants were instructed that the empty circle appear randomly between correct trials.

## Results and discussion

Similarly to Experiment 1, Here too errors, post-error trials (total of 17.4%) as well as trials with outlier RTs (+/- 2 standard scores and under 300 ms; total of 2.97%) were excluded from the performance RT analysis. The prevalence of each feedback stimulus across participants was almost identical here too with 23.107% (n = 1117) gain trials, 23.065% (n = 1115) neutral trials, 22.527% (n = 1089) neutral non-feedback trials, and 22.527% (n = 1089) sad trials.

### Effects of gain and loss on arithmetic performance

**Fig 4:**
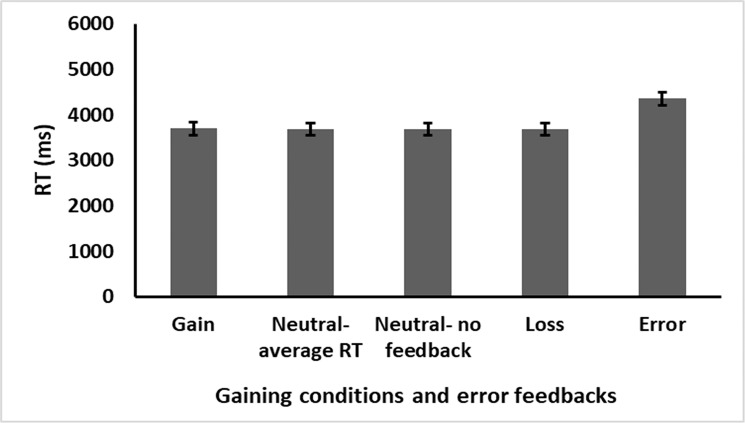
Experiment 2 results. Performance after the different gaining conditions. Error bars represent standard error.

A similar three-way ANOVA as in Experiment 1 was applied here as well with RT (*n+1*) as the dependent variable. A significant effect was found for equation difficulty, *F*(1,21) = 217.317, *p* < .001, η_p_^2^ = 0.912, and correctness, *F*(1,21) = 9.393, *p* = .012, η_p_^2^ = 0.309, suggesting participants solved the more difficult three addends equations and incorrect equations slower than the easier two addend and correct ones. Furthermore, the interaction between equation difficulty and correctness was found significant as well, *F*(1,21) = 34.821, *p* < .001, η_p_^2^ = 0.624. Again, here too the gaining condition (i.e., gain, neutral feedback, neutral non-feedback, or loss) yielded no significant results, *F* < 1, suggesting no differences in RT between the different gaining conditions (see Fig 4). No other main effects or interactions were found, all *p*s > .05. Examining the accuracy rate under the different gaining conditions resulted in no significant effects *F* < 1.

Similar to Experiment 1, here too we undertook a Bayesian statistical approach aimed at exploring whether the non-significant results regarding RT in the gaining condition is a result of the true null hypothesis. The analysis revealed a BF_10_ of 0.012 (considered strong evidence for the null hypothesis suggesting no differences between the different gaining conditions). Further again, we used the Dienes online calculator [76] with the effect from the previous study as priors (same priors as in Exp 1) in order to specifically compare the gain and loss stimuli. The results yielded a BF_10_ of 0.15 (95% HDI = 0.007–0.481). This BF value is considered moderate support for the null hypothesis suggesting no differences in RT after gain when compared to after loss stimuli.

### PES in arithmetic performance

Here too, we analyzed the post-error effects on performance and therefore again included post-error trials. Here, the prevalence of post-errors was 8.2% (n = 433) across all trials. Participants solved arithmetic equations significantly slower after errors when compared to correct trials, *t*(21) = 3.161, *p* = .005, *d* = 0.674, BF_10_ of 9.404 (considered moderate support for the alternative hypothesis). Accuracy did not differ depending on the previous trials’ accuracy (i.e., previous incorrect versus previous correct), *t*(21) = 0.964, *p* = .346, *d* = 0.205. BF_10_ of 0.337 (considered moderate support for the null hypothesis).

### Comparing the effect of errors versus aversive stimuli on arithmetic performance

The same analysis as in Experiment 1 was applied to the data here as well. RT differed between the different feedback conditions, *F*(4,84) = 16.637, *p* < .001, η_p_^2^ = 0.442, BF_10_ of 1.864e+7 (considered extreme support for the alternative hypothesis). Further analysis revealed that participants solved arithmetic equations significantly slower after errors when compared to loss stimuli, *t*(21) = 4.604, *p* < .001, *d* = 0.982, BF_10_ = 187.4, neutral feedback stimuli, *t*(21) = 4.984, *p* < .001, *d* = 1.062, BF_10_ = 419.3, and neutral non-feedback stimuli, *t*(21) = 4.849, *p* < .001, *d* = 1.034. BF_10_ = 314.9. (Note that all these BF values represent extreme support for the alternative hypothesis.).

To sum up, our findings from Experiment 2 replicate the results of Experiment 1 suggesting that when perceived as performance-contingent, gain stimuli did not differentially affect arithmetic performance when compared to loss. Furthermore, here too, performance after errors led to slower performance both when compared to after correct trials or other negatively salient feedbacks.

## Joint analysis

In order to directly compare the current results with the findings obtained in the former study by Naaman and Goldfarb [15], another statistical analysis was conducted comparing the data from the previous random contingency experiment to the current performance-contingent ones. For that matter, the pooled data of the effect of gains versus losses across both contingency types was analyzed using a repeated measures ANOVA with the administration contingency (random versus performance-contingent) as the between subject independent variable, the gaining condition (gains versus losses) as the within subject independent variable and performance RT as the dependent variable. Finally, we conducted an equivalent Bayesian analysis on the same variables.

According to the joint analysis, participants performed faster after gains when compared to loss *F*(1,84) = 10.742, *p* < .01, η_p_^2^ = 0.113, BF_10_ of 2.147. No differences in RT observed between random and performance-contingency administration settings *F*(1,84) = 1.893, *p* = .172, η_p_^2^ = 0.021, BF_10_ of 0.504. Of interest, the interaction between administration contingency and gaining condition was significant *F*(4,84) = 3.087, *p* < .05, η_p_^2^ = 0.128, BF_10_ of 9.914. further analysis of the interaction between administration contingency and gaining condition revealed that participants performed faster after gains when compared to after loss under the random administration settings *t*(41) = 3.626, *p* < .001, *d* = 0.559, BF_10_ of 37.18 but not under the performance-contingent administration settings *t*(46) = 0.558, *p* = .710, *d* = 0.081, BF_10_ of 0.108.

## General discussion

The current study examined the effects of errors and perceived performance-contingent feedback on arithmetic performance. In two experiments we found that when perceiving gain and loss as performance-contingent, the modulation of arithmetic performance, seen previously under random contingency conditions [15, 42], was entirely eliminated. In addition, we found that post-error performance was in fact slower when compared to post-correct performance (i.e., PES), as previously reported [50, 58, 63, 66]. Furthermore, we found that only errors, but not other aversive feedback, modulated performance in the subsequent trial. Finally, we compared the current findings on perceived performance-contingent feedbacks to the previous findings of Naaman and Goldfarb on the random feedbacks design [15]. The joint analysis revealed an interaction between the administration contingency and gaining condition resulting from a facilitating effect of gains on performance but only under the random contingency settings.

### The modulation of performance-contingent gain and loss on arithmetic performance

As discussed above, while the dissociation between random and performance-contingent gain/loss is well documented, its direction seems to differentiate across different studies and experimental designs [17, 21, 26, 28, 29, 36]. Specifically, according to the model suggested by Braem and Notebaert described above, while abstract reward signals better relate to motivational aspects and thus enhance attentional focus (e.g., cognitive stability), other more salient reward stimuli (e.g., emotional pictures) better relate to the hedonic aspect of reward and thus enhance a more exploratory mode, resulting in better cognitive flexibility [36, 39, 40].

In the context of arithmetic performance, only two previous studies examined the influence of gain and loss stimuli on performance, both in a random stimuli administration environment [15, 42]. Interestingly, both found performance facilitation after the presentation of random gains (when compared to loss stimuli). It is important to note that while both the current study and the previous study by Naaman and Goldfarb used a similar random stimuli administration method, participants in the current study perceived the stimuli administration as performance-contingent. This change in contingency perception was enough to completely diminish the facilitative effect found under random stimuli administration conditions. Furthermore, we directly compared both contingencies by analyzing the pooled data from both the current and the previous study by Naaman and Goldfarb [15]. The findings from the joint analysis revealed a significant interaction between the administration contingency and gaining condition according to which, the observed differences in RT after gains compared to after losses only occurred under the random contingency settings but not under the performance-contingent settings.

The current pattern of results is in line with the findings of Gable and Harmon (Experiment 2) described above [36]. Both studies present a similar pattern, in which, while random gains lead to a facilitative effect on performance as represented by faster RTs, performance-contingent gains do not result in a similar difference in performance. Note, that while Gable and Harmon examined the effects of gain cues and feedbacks on lexical decisions, flanker, and Navon tasks, we examined this issue in arithmetic performance. Examining these effects specifically in the arithmetic domain is of great importance, since generalizing previous findings from other domain areas (e.g., conflict monitoring tasks or lexical tasks) might prove misleading. Furthermore, the literature on this issue portrays differentiation in pattern and directionality across different domain areas [17, 28, 29, 80]. This differentiation might suggest that the difference between arithmetic and other cognitive tasks can result in entirely different pattern of gain and loss modulation of performance.

Considering Braem and Notebaert’s theoretical framework discussed above [39], while random gain stimuli might have tapped the hedonic component of reward, resulting in a mode of enhanced cognitive flexibility [40], performance-contingent rewards tapped the "learning" component of reward, resulting in a mode of enhanced cognitive stability [17, 28, 29, 39]. Importantly, while smiley faces might be considered as relating to the hedonic aspects of reward according to the model, in the current study they were attached to small monetary gains and losses and administered in a perceived performance-contingent manner and therefore strongly relate to the learning component of reward.

In the context of the current findings, it is important to note that cognitive flexibility, alongside other EF, is suggested to play an important role in arithmetic calculations. A growing amount of data supports the relationship between flexibility and arithmetic performance [7, 81–83]. Furthermore, children with mathematic difficulties were found to exhibit poorer ability in switching between retrieval strategies compared to controls [84]. While the exact role of cognitive flexibility in arithmetic performance is not fully understood, it has been suggested to be involved in efficiently switching between solution strategies, arithmetic operations, and calculation steps in complex multistep arithmetic [85, 86]. For example, cognitive flexibility was found to be a key factor in maintaining a diverse repertoire of solution strategies and in efficiently selecting the best solution strategy required for different arithmetic problems [83].

The arithmetic task in the current study included calculation of two and three addend non-carry equations. In non-carry equations, the sum of each position (units, tens, hundreds, etc.) of all respective addends never exceeds 9 (23+46 or 21+35+42). Consequently, solving three addend non-carry equations obligates calculation and maintenance of intermediate sums, resulting in multiple step calculation that requires efficient switching between calculation stages, among other higher cognitive demands (e.g., WM and inhibition). On the other hand, two addend non-carry equations are assumed to be associated with less cognitive effort. For example, when calculating 31+47 no calculation of intermediate sums is required, but when calculating 15+42+21 the holding and processing of the intermediate sum of the first two addends (e.g., 15+42 = 57) is obligated in order to add it to the third addend and calculate the final solution. Thus, performing three addend non-carry equations requires more complex multiple step processes and the involvement of EF (i.e., W.M and inhibition) to a greater extent than in two addend equations. This shift between two and three addend equations and between multiple calculational steps requires flexibility in order to efficiently switch between different calculation steps and solution strategies. To sum, it is possible that whereas random gains promoted flexibility, required for the efficient calculation and strategy switch in the arithmetic task, performance-contingent gains did not promote flexibility and therefore did not result in better performance when compared to after-loss stimuli.

It is worth noting that the current study used perceived performance contingency method and relatively small sample of participants. Using perceived contingency holds a great methodological advantage since it enables direct comparisons between these two contingency paradigms (i.e., random versus performance contingencies) under a similar experimental design and manipulation only on the participants’ perception. While the current conclusions are based on perceived performance contingency, future studies could examine this issue using a true performance-contingent design, based on actual participants’ performance. While the use of true contingency methods will not allow direct and clean comparisons to random contingencies, it will broaden our knowledge and understanding with regarding to performance-contingent feedbacks. Furthermore, future replication of the current study with a larger sample size is needed in order to strengthen our findings and further support our conclusions.

### The influence of error versus negative feedback on arithmetic performance

While gain and loss feedback did not affect arithmetic performance, error feedback did result in PES in the subsequent trial. Specifically, performance after error feedbacks resulted in significant increase in RT (i.e., PES) even when compared to other negative performance feedback such as loss (represented by sad faces). This is the first study to examine all these types of feedbacks together in the arithmetic context.

In addition, by comparing error feedback to other negative valence feedback, our findings shed new light on the influence of these two types of signals in the context of arithmetic performance. Specifically, our findings suggest that error signals seem to have a more pronounced influence on arithmetic performance than other types of negative or positive performance signals. Interestingly, this more pronounced influence was found even though error feedbacks, as opposed to performance RT feedback (i.e., gain and loss stimuli) had no direct monetary meaning. That is, while the other performance feedbacks (e.g., sad or smiley faces) were accompanied by certain monetary meaning, participants were instructed that errors would not have monetary consequence. Nevertheless, the effect of errors on performance was substantially more pronounced when compared to the other monetary coupled feedbacks.

As noted above, it has been suggested that errors are assumed to represent emotionally and motivationally salient events and that their aversive signals activate the defensive motivational systems that modulate behavior accordingly as post-error adjustments [43, 46, 52–54]. However, the current findings suggest that negative emotion or motivation are not the only factors determining the influence on arithmetic performance and that errors might represent unique signals that influence our arithmetic performance. This is in line with studies in other domains that found that errors have a unique influence in different tasks [29, 39, 87].

During our everyday life, we encounter numerous situations in which we perceive performance feedback in the form of errors, rewards, punishments, gains, and losses. These are also seen in situations of arithmetic performance, both within our formal education (e.g., receiving feedback for performing arithmetic procedures in a test or in front of the classroom) and outside it (e.g., dividing the bill between friends in a restaurant). Therefore, it is of great importance to better examine and differentiate these situations from one another, as well as differentiating between situations that are perceived as performance-contingent or random. The current study further broadens previous findings regarding the influence of errors, gains, and losses on arithmetic performance. It sheds new light and expands our understanding in the context of a performance-contingent environment. The presence of both random and performance-contingent perceived cues in our environment is considerably large. Together with the critical role of arithmetic in our daily life, studying this affective cognitive interaction between the two requires more scientific attention, as it might influence how we learn and perform arithmetic.

## Supporting information

S1 Data EXP1 RT after errors vs other feedbacks. (XLSX)

S2 Data Exp1 RT ANOVA. (XLSX)

S3 Data EXP2 RT after errors vs other feedbacks. (XLSX)

S4 Data Exp2 RT ANOVA. (XLSX)
